# Attitudes toward osteopathic medicine scale: development and psychometrics

**DOI:** 10.5116/ijme.615c.2cfa

**Published:** 2021-11-19

**Authors:** Mohammadreza Hojat, Jennifer DeSantis, Robert A. Cain, Mark R. Speicher, Lynn Bragan, Leonard H. Calabrese

**Affiliations:** 1 Asano-Gonnella Center for Research in Medical Education and Health Care, Sidney Kimmel Medical College at Thomas Jefferson University, Philadelphia, Pennsylvania, USA; 2 American Association of Colleges of Osteopathic Medicine, Bethesda, Maryland, USA; 3 Cleveland Clinic Lerner College of Medicine of Case Western Reserve University, Cleveland, Ohio, USA

**Keywords:** Osteopathic medicine, attitudes, psychometric, empathy, burnout, lifelong learning, interprofessional collaboration

## Abstract

**Objective:**

To develop a valid and reliable instrument for measuring attitudes toward osteopathic medicine.

**Methods:**

Participants included 5,669 first-year students from 33 U.S. colleges of osteopathic medicine, who completed an online survey at the beginning of the 2019-2020 academic year. Using data from the nationwide Project in Osteopathic Medical Education and Empathy, we developed a 13-item instrument: Attitudes Toward Osteopathic Medicine Scale (ATOMS) and demonstrated the validity and reliability of its scores. The social desirability response bias was controlled in statistical analyses.

**Results:**

The corrected item-total score correlations were all positive and statistically significant, and the effect sizes of item discrimination indices were large. Cronbach’s coefficient alpha reliability was 0.83. Construct validity, corroborating face and content validity of the ATOMS, was supported by three components, emerged from factor analysis: “Perspectives on Osteopathic Medicine,” “Osteopathic Diagnosis and Treatment,” and “Holistic-Integrative Care.” Correlations between ATOMS scores and scores of cognitive empathy, emotional empathy; orientation toward interprofessional collaboration; lifelong learning; and burnout were statistically significant in the expected direction, providing validity evidence for the ATOMS. Using the method of contrasted groups, significant differences in the ATOMS scores were found by gender, ethnicity, academic background, and career interest in the expected direction, supporting the validity of the ATOMS scores. National norms were developed to assess individual scores alongside national percentile ranks.

**Conclusions:**

The ATOMS, developed in a nationwide study, supported by strong psychometric evidence for measuring orientation toward osteopathic medicine, has implications for the assessment of osteopathic medical education, patient outcomes, and admission decisions.

## Introduction

Diagnosis and treatment of illness in the context of holistic care was recognized in 1874 by Andrew Taylor Still, MD, who called his view of medical care “osteopathy” and founded the first osteopathic medical school in 1892 in Kirksville, Missouri (currently A.T. Still University-Kirksville College of Osteopathic Medicine). The core tenets of osteopathic medicine specify that a human is a unit of the physical, mental, and social/spiritual; that the body is capable of self-regulation; and that holistic treatment should be based upon an understanding of body unity, self-regulation, and interrelationships of structure and function.^1^^,^^2^

Fundamental osteopathic medical competencies include the application of osteopathic manual diagnosis and treatment; the ability to work effectively with other health care professionals as members or leaders of an interprofessional collaborative team; and demonstration of humanistic behavior such as empathy, altruism, compassion, respect, integrity, honesty, and trustworthiness.^3^ While osteopathic and allopathic medical education systems currently share most of the aforementioned features in educating physicians-in-training, osteopathic medicine emphasizes manipulative diagnosis and treatment and holistic care.

Attitudes toward specific features and tenets of osteopathic medicine contribute to the career decisions of applicants and to the practice of medicine by graduates of osteopathic medical schools. Also, the measurement of such attitudes with a psychometrically sound instrument would be crucial for the assessment of osteopathic medical, educational outcomes. However, while reviewing the relevant literature, we noticed a limitation in empirical research in identifying core components of the attitudes toward osteopathic medicine. There are a few instruments intended to measure attitudes, orientation, and beliefs toward aspects of osteopathic medicine, such as osteopathic principles,^4^ osteopathic manipulative treatment, osteopathic philosophy,^5^^-^^8^ and osteopathic education.^9^ However, these instruments have not been supported by strong psychometric, especially validity evidence. Study participants were often accessible samples from a single institution and insufficient in size.

There was a need for a valid and reliable instrument, developed without suffering from the aforementioned limitations, for measuring attitudes toward osteopathic medicine. In response to this need, we designed this study to develop a psychometrically sound instrument for measuring empirically derived aspects of osteopathic medicine, with potential implications for the assessments of osteopathic medical education outcomes and clinical outcomes of osteopathic care, and to monitor changes as physicians-in-training progress through medical school and postgraduate medical education training. Also, our intention from the onset of the study was to use nationwide data to provide a national norm table for osteopathic medical students to assess their scores on the instrument against the national norm, and possibly use each osteopathic medical school applicant’s converted national percentile rank as a supplementary measure for admission decisions.

### The nationwide project

This study is part of the landmark nationwide Project in Osteopathic Medical Education and Empathy (POMEE), a two-phased project sponsored by the American Association of Colleges of Osteopathic Medicine and cosponsored by the American Osteopathic Association, in collaboration with the Cleveland Clinic and Sidney Kimmel Medical College at Thomas Jefferson University. Phase I, a 2-year cross-sectional study completed in 2018, laid the foundation for Phase II, a 5-year longitudinal study of changes in empathy and other personal qualities, including attitudes toward osteopathic medicine as students progress through medical school. Data for this article were retrieved from the database of POMEE-Phase II.

## Methods

### Research design and study cohort

Participants in this survey research included 5,669 first-year matriculants to 33 of 36 (92%) of U.S. colleges of osteopathic medicine in the 2019-2020 academic year who voluntarily participated in the study.

The research team at Jefferson obtained exempt status approval for the project from Thomas Jefferson University Institutional Review Board (IRB); all other participating colleges also received IRB approval from their college.

### Study survey

The study survey included questions regarding participants’ demographics, undergraduate major, and career interest, plus the following instruments:

#### Attitudes Toward Osteopathic Medicine Scale

We developed a new instrument: Attitudes Toward Osteopathic Medicine Scale (ATOMS); seven items of this instrument were adapted (with author permission) from a 29-item Integrative Medicine Attitudes Questionnaire;^10^ six items were developed by two of this study’s authors (MH and LHC) for another study with osteopathic medical students.^11^ Permission to use selected items from the Integrative Medicine Attitudes Questionnaire was obtained from the author of the questionnaire. Items were answered on a 7-point Likert scale (1=Absolutely Disagree, 7=Absolutely Agree).

#### Cognitive (Clinical) Empathy

We measured clinical empathy using the Jefferson Scale of Empathy (JSE, 20-item, medical student version), a broadly used and validated instrument for measuring clinical empathy in the context of patient care, developed based on the conceptualization of empathy as a predominantly cognitive attribute. Evidence from medical school student samples in the U.S. and abroad supports psychometrics of the JSE^12^  and specifically in first-year matriculants of osteopathic medical schools (POMEE Phase I).^13^ Moreover, the JSE has been recognized as the most studied instrument in medical education research,^14^ and the most frequently used instrument for measuring clinical empathy in medical education.^15^

#### Emotional Empathy

Cognitive and emotional empathy could have different consequences in the context of patient care.^12^ Because of its affective nature, excess emotional empathy (synonymous with sympathy) can be detrimental to patient care.^12^ We included emotional empathy to differentiate the effects of cognitive empathy (understanding patient’s suffering) from emotional empathy (e.g., feeling patient’s pain) on outcome measures. We selected the following two scales from the Interpersonal Reactivity Index (IRI): “empathic concern” and “personal distress.”^16^^-^^17^ Each scale contains seven items. The total score of these two scales was considered as an indicator of emotional empathy.^16^^-^^18^ Moderate correlations between scores of the IRI scales and the JSE have been reported in medical students.^19^ Permission to use this instrument in this study was obtained from the author of the IRI.

#### Attitudes Toward Interprofessional Collaboration

We used the Jefferson Scale of Attitudes Toward Interprofessional Collaboration (JeffSATIC), a 20-item validated instrument measuring orientation toward interprofessional collaboration and teamwork in health professions students and practitioners. Evidence supporting measurement properties of this instrument has been reported in a multi-institutional and multi-national study of health professions students.^20^

#### Attitudes Toward Lifelong Learning

We used the Jefferson Scale of Attitudes Toward Physician Lifelong Learning (JSPLL), a 14-item instrument adapted from the Jefferson Scale of Physician Lifelong Learning,^21^ for administration to medical students.^22^ Evidence has been reported supporting measurement properties of the JSPLL in physicians^21^ and medical students.^22^

#### Burnout Measure

We used the Shirom-Melamed Burnout Measure (BM),^23^^-^^25^ a 14-item instrument to measure overall burnout experiences. The instrument has been used in a multi-institutional study with medical students.^26^ Permission to use this instrument in this study was obtained from its author.

#### “Good Impression” Response Bias

Respondents to self-reported personality tests can manipulate their answers to produce disingenuous responses, known as the “social desirability response set.” We used the “Infrequency” Scale of the Zuckerman-Kuhlman Personality Questionnaire (ZKPQ)^27^ to control for the effect of the social desirability response bias. This 10-item scale identifies subjects with invalid records due to an exaggerated “good impression” response bias. Scores higher than three on this scale indicates questionable validity of the respondent record.^27^ This scale was previously used with medical students to detect and control for the tendency to make “good impression” responses,^12^^,^^28^ and in POMEE-Phase I.^13^^,^^29^^-^^31^

### Procedures

Two pilot studies were undertaken with volunteer osteopathic medical students and medical education researchers to improve the clarity and comprehensiveness of the study survey, to detect any possible technical issues in its online administration (pilot study 1), and to test it when using desktop, laptop, and mobile devices (pilot study 2).

One or two research coordinators from each participating college or campus were selected to serve as liaisons between students, the colleges, AACOM, and the research team at Jefferson. Research coordinators and the AACOM research team arranged with college administrators to schedule an appropriate time for online group administration of the study survey at local campuses and helped to maximize response rates.

Participants were informed that their email addresses would be used as a unique identifier to track, match, and merge data from multiple survey administrations. Before the administration of the survey, students received an email signed by the dean of their medical school that included a brief message about the importance of the project and its goals. Subsequently, students received another email message, encouraging them to participate as an “indispensable” stakeholder of this landmark project, signed by Robert Cain, DO, president and CEO of the AACOM, and Leonard Calabrese, DO, of the Cleveland Clinic (principal co-investigators of the project).

We administered the initial web-based study survey at the beginning of the 2019-2020 academic year prior to the start of medical school classes. Our study survey accompanied the AACOM matriculating student survey. Respondents were given the option to voluntarily complete the accompanying study survey. They could also voluntarily enter their email addresses to receive feedback on their empathy scores. Online administration of the study survey was managed by the AACOM research team.

### Statistical analyses

We calculated Cronbach’s coefficient alpha, and examined corrected item-total score correlations, item discrimination effect sizes, underlying factor structure, and used bivariate correlations (Pearson), multivariate regression analysis, and the method of contrasted-groups to confirm the validity and reliability of the ATOMS, developed in this study. The Statistical Analysis System (SAS for Windows, version 9.4) was used for statistical analyses.

## Results

A total of 5,979 students of 7,781 total first-year matriculants in all U.S. colleges of osteopathic medicine (77%) submitted their online survey. Excluded were incomplete surveys and respondents’ records with questionable validity (scored>3 on the Infrequency Scale of the ZKPQ). Therefore, the final sample for statistical analyses included 5,669 students; 2,653 self-identified as male (47%); 2,964 (52%) as female; and 52 (< 1%) did not identify as either male or female.

### Preliminary Study of the ATOMS

We performed a preliminary study to examine corrected item-total score correlations and explore underlying factors of the initial instrument (14-item). The corrected item-total score correlations were all positive and statistically significant with the exception of one item that read: “A patient is healed when the underlying pathological processes are corrected or controlled”, for which the item-total score correlation was negative and negligible (r=-0.10), with a non-substantial factor loading. We deleted this item; thus, the final ATOMS contained 13 items used for further statistical analyses (see Appendix A).

### Item-Total Score Correlations

The corrected item-total score correlations of the final 13-item ATOMS instrument (calculated based on the correlation between each item score and total score, excluding the corresponding item from the total score) were statistically significant and moderately high, ranging from a low of 0.29 (p< 0.01) for this item: “Therapeutic touch has been discredited as a healing modality” (a reverse-scored item) to a high of 0.61, p<0.01) for this item: “Osteopathic manipulative therapy is a valuable method for resolving a wide variety of musculoskeletal problems”. The median correlation was 0.49 (Table 1).

### Effect Sizes of Item Discrimination Indices

The effect sizes of item discrimination indices were calculated by subtracting the item mean score for the top 33% ATOMS scorers from the mean score of the same item obtained by the bottom 33% ATOMS scorers, divided by the pooled standard deviation of the corresponding item (Table 1). These effect sizes were analogous to Cohen’s d statistics.^32^ All of the effect sizes were substantially large (> 1.01).

### Exploratory Factor Analysis

We examined the underlying construct of the 13-item ATOMS by conducting exploratory factor analysis, using principal components with oblique (promax) rotation to allow correlations among the factors (Table 1). Three factors emerged, each with an eigenvalue greater than 1 (Kaiser Criterion). The eigenvalues before rotation were 4.41, 1.59, and 1.04, and accounted for 34%, 12%, and 8% of the total variance, respectively. The scree test showed that the plot of eigenvalues leveled off after the third extracted factor, supporting the retention of the three factors. The Kaiser-Meyer-Olkin measure of sampling adequacy (MSA) showed an overall index of 0.88, indicating that data were adequate for factor analysis. Bartlett’s test for sphericity indicated that the intercorrelation matrix was factorable (χ^2^_(42)=_463.82, p<0.0001).

The first factor was entitled “Perspectives on Osteopathic Medicine” (rotated factor loadings ≥ 0.42 in its five items). A typical item representing this factor is: “A strong relationship between patient and physician is an extremely valuable therapeutic intervention that leads to improved outcomes.” The second factor, “Osteopathic Diagnosis and Treatment”, included five items with factor loadings ≥ 0.46. A typical item representing this factor is: “Touch and tactile approaches may not serve a significant purpose in patient care” (a reverse-scored item). The third factor, “Holistic-Integrative Care”, included three items with factor loadings ≥ 0.54. A typical item representing this factor is: “The osteopathic philosophy of holistic care greatly influenced my decision to attend an osteopathic school.” The Cronbach’s coefficient alphas for the three extracted factors were 0.77, 0.71, and 0.73, respectively.

### Descriptive Statistics

The obtained mean and standard deviation of ATOMS scores were 73.9 and 9.5, respectively; the possible and actual score ranges were 13-91 and 29-91, respectively.

### Criterion-Related Validity

We examined bivariate Pearson correlations between scores on the ATOMS and those of other personal quality measures used in the study (Table 2). All obtained correlations were statistically significant, ranging from highs of 0.60 (p<0.01) for interprofessional collaboration, and 0.58 (p<0.01) for clinical empathy, to a low of 0.17 (p< 0.01) for emotional empathy. Correlation between scores of the ATOMS and burnout measure was statistically significant and negative (r= -0.29, p< 0.01).

We performed multiple regression analysis to examine the unique contribution of each of the personal quality measures in predicting scores on the ATOMS. Table 2 shows standardized regression coefficients (β), unstandardized regression coefficients, standard errors, t-values, and statistical significance for the unique contributions of the regressors in predicting ATOMS scores.

Measures of interprofessional collaboration (β=0.33), and clinical empathy (β=0.30) provided the most unique and positive contributions to predicting ATOMS scores in the multivariate model, and orientation toward lifelong learning (β=0.13) and emotional empathy (β=0.08) provided the least. The burnout measure showed a statistically significant negative contribution. The adjusted multiple correlation was R=0.68, meaning that 46% (R^2^=0.68^2^=46%) of the variation in the ATOMS scores could be accounted for by the five regressors (Table 2).

In the additional analysis, we found a significant inverse association between clinical (cognitive) empathy and burnout (r=-0.21, p <0.01), whereas the correlation between emotional empathy and burnout was positive (r=0.14, p<0.01). This pattern of finding was expected as described in the discussion of findings.

### Validity Evidence by the Method of Contrasted Groups

Significant differences have been found on scores on the JSE and gender (in favor of women),^12^^,^^31^ and on ethnicity (in favor of African-American and Latinx vs Asian-American and White medical students),^31^   academic background (in favor

**Table 1:** Rotated Factor Loadings(a), Corrected Item-Total Score Correlations(b), and Effect Sizes of Item Discrimination Indices(c) for the Attitudes Toward Osteopathic Medicine Scale (ATOMS) in a National Sample of First Year Matriculants of 33 U.S. Colleges of Osteopathic Medicine

Item	Factor1	Factor2	Factor3	Corrected item-total score correlation	Item discrimination index effect size
Physicians who strive to understand themselves provide better care than those who do not. (10)	0.75	0.00	-0.02	0.51	1.40
Physicians who model a balanced lifestyle (i.e., Attending to their own health, social, family and spiritual needs, as well as interests beyond medicine) generate improved patient satisfaction. (4)	0.68	-0.02	0.04	0.49	1.25
A strong relationship between patient and physician is an extremely valuable therapeutic intervention that leads to improved outcomes. (6)	0.65	0.08	0.02	0.54	1.44
Psychosocial factors are as important as biomedical factor in health and illness. (11)	0.54	0.09	0.04	0.47	1.25
Instilling hope in patients is a physician’s duty. (7)	0.42	-0.11	0.13	0.31	1.02
Medical problems need specific medical and surgical interventions, thus, holistic approaches to medical problems cannot be as beneficial as targeted biomedical treatment. (12)	0.05	0.67	-0.05	0.48	1.56
Touch and tactile approaches may not serve a significant purpose in patient care. (13)	0.08	0.62	0.03	0.54	1.55
Osteopathic Manipulation often makes patients “feel” better temporarily but does not lead to objective improvement in long-term outcomes for patients. (5)	-0.12	0.52	0.32	0.54	1.67
Information about the relative effectiveness of treatments that is obtained by research methods other than randomized controlled trials has little value to physicians. (9)	-0.02	0.48	-0.03	0.32	1.15
Therapeutic touch has been completely discredited as a healing modality. (3)	-0.01	0.46	-0.05	0.29	1.09
Osteopathic manipulative therapy is a valuable method for resolving a wide variety of musculoskeletal problems (beyond back pain). (8)	0.07	0.06	0.71	0.61	1.72
The osteopathic philosophy of holistic care greatly influenced my decision to attend an osteopathic school. (1)	0.05	0.00	0.66	0.51	1.42
Patients whose physicians are knowledgeable of multiple medical systems and complementary and alternative practices, in addition to conventional medicine, do better than those whose physicians are only familiar with conventional medicine. (2)	0.18	-0.06	0.54	0.49	1.45
Eigenvalue	4.41	1.59	1.04		

^(a) ^Based on the content of items with high factor loadings, Factor 1: was entitled “Perspectives on Osteopathic Medicine”, Factor 2: “Osteopathic Diagnosis and Treatment”, and Factor 3: “Holistic-Integrative Care”.   Items are sorted by descending order of factor loadings within each factor. Number in parentheses refer to the appearance of the items in the ATOMS.

^(b) ^Correlations between scores on each item and the ATOMS total score by excluding the corresponding item from the total score, all were statistically significant (p< 0.01).

^(c) ^Effect size estimate (Cohen’s d statistic) of the discrimination index was calculated by subtracting the item mean score of the ATOMS high scorers (top 33%) from the item mean score of the ATOMS low scorers (bottom 33%), divided by the pooled standard deviation of the corresponding item.

of those with undergraduate college majors (in favor of those with college majors in social and behavioral sciences, and arts and humanities)^31^ and career interest (in favor of medical students who planned to pursue “People-Oriented” specialties such as general internal medicine, family medicine, pediatrics, and psychiatry versus others interested in “Technology/Procedure-Oriented” specialties such as pathology, anesthesiology, radiology, and surgery.^12^^,^^31^ Because of significant and relatively large correlations we observed in this study between the ATOMS and JSE scores, we expected to similarly find significant differences on scores of the ATOMS by gender (in favor of women), ethnicity (in favor of African-American and Latinx), academic background (in favor of those with college majors in social and behavioral sciences, arts and humanities), and career interest (in favor of those planning to pursue “People-Oriented” specialties. Using analysis of variance, we examined group differences on the ATOMS scores by gender, race/ethnicity, academic background, and career interest to find out if group differences were in the expected direction. Means, standard deviations, and summary results of statistical analyses are reported in Table 3.

### Gender Difference

The ATOMS mean score for men was 71.5 (SD=10.0), and for women was 76.1 (SD=8.3). Gender difference in favor of women was statistically significant (F_(__1,5615)_=362.31, p< 0.0001). The difference was also practically important, as indicated by the effect size of 0.51.

**Table 2:** Summary Results of Multiple Regression Analysis(a) in a National Sample of First Year Matriculants of 33 U.S. Colleges of Osteopathic Medicine

Regressors	Bivariate correlation^(b)^	Standardized regression coefficient (β)^(c)^	Unstandardized regression coefficient^(d)^	Standard error	t-value
JSE	0.58^**^	0.30	0.25	0.01	23.00^**^
IRI	0.17^**^	0.08	0.13	0.02	7.50^**^
JeffSATIC	0.60^**^	0.33	0.25	0.01	25.08^**^
JSPLL	0.36^**^	0.13	0.22	0.02	11.73^**^
BM	-0.29^**^	-0.12	-1.27	0.11	-11.57^**^
Intercept	-	0	3.62	1.33	2.73^*^

^**^p<0.0001, ^*^p<0.05, adjusted multiple R = 0.68, p<0.0001

^(a) ^Scores on the Attitudes Toward Osteopathic Medicine Scale (ATOMS) as the dependent variable, and personality measures as regressors.

^(b)^ Bivariate correlation between the ATOMS scores and other personality measures shown in the table.

^(c)^Standardized regression coefficients (β) are calculated from score distributions converted to a standard distribution for all regressors with a mean of zero and standard deviation of 1 for comparability purposes.

^(d)^Unstandardized regression coefficients are calculated from raw score distributions.

Abbreviations: JSE: Jefferson Scale of Empathy; IRI: Interpersonal Reactivity Index; JeffSATIC:  Jefferson Scale of Attitudes Toward Interprofessional Collaboration; JSPLL: Jefferson Scale of Physician Lifelong Learning; BM: Shirom-Melamed Burnout Measure.

### Race/Ethnicity Differences

The highest mean score on the ATOMS was obtained by Black/African American students (M=77.27, SD=9.2), and the lowest by Asian students (M=72.88, SD=9.5). The mean scores of the White and Hispanic/Latinx/Spanish origin groups were in between the other two groups. The differences in favor of the African/American group versus Asian, White, and Hispanic/Latinx/Spanish origin groups were statistically significant (F_(__3,5099)_=15.80, p<.0001). Also, Hispanic/Latinx/Spanish origin groups obtained mean scores that were significantly higher than those obtained by White and Asian groups. The race/ethnic differences were practically important (effect size between the highest and lowest scoring groups=0.47).

### Academic Background

Respondents were asked to report their undergraduate major by choosing from a list of 56 undergraduate majors (sorted alphabetically). For statistical analysis, we grouped the undergraduate majors into the following four broad categories: “Biological Sciences,” “Chemical/Physical Sciences,” “Social/Behavioral Sciences,” and “Arts and Humanities.” We compared respondents with different undergraduate majors on ATOMS scores.

The majority reported their undergraduate degree in “Biological Sciences” (n=2,833), followed by those who majored in “Chemical/Physical Sciences (n=755), “Social/Behavioral Sciences” (n=264), and “Arts and Humanities” (n=91). The lowest ATOMS mean score was obtained for those who majored in “Chemical/Physical Sciences” (M=71.8, SD=9.7), which was significantly lower than the scores in the other three academic background groups (F_(__3,3939)_=13.04, p< .0001).

### Career Interest

Respondents were asked to choose the specialty they planned to pursue after graduation from medical school from a list of 33 specialties, most frequently pursued by graduates of colleges of osteopathic medicine. Based on other studies with allopathic^33^ and osteopathic medical students,^31^ we divided the specialties into three broad categories: “People-Oriented” (e.g., family medicine, internal medicine, obstetrics and gynecology, and pediatrics); “Technology-/Procedure-Oriented” (e.g., anesthesiology, dermatology, ophthalmology, orthopedic surgery, radiology, and surgery); and “Other” (including specialties chosen by fewer than 20 matriculants).

Summary results of statistical analyses are reported in Table 3. The highest ATOMS mean score was obtained by those planning to pursue “People-Oriented” specialties (M=75,17, SD=9.0), and the lowest by those planning to pursue “Technology-/Procedure-Oriented” specialties (M=72.55, SD=9.9) (F_(__2,5110)_=40.21, p<0.0001).

### National norms

Using a national sample in this study provided a unique opportunity to develop national norms for the ATOMS scores that will enable medical colleges to determine the percentile rank of any new matriculant to osteopathic medical schools. Because of the gender difference in ATOMS scores observed in this study, we calculated percentile ranks for men and women separately (Table 4). For example, if the ATOMS score of a male matriculant is 80, first find the score interval that includes a score of 80 (79-80 score interval in Table 4), then find the corresponding national percentile rank displayed in the row for that score interval in the table. The corresponding national percentile rank in the table for a male matriculant with an ATOMS score of 80 is 78%, meaning that a score of 80 places a male matriculant in the 78th percentile rank of all first-year male matriculants. However, a female matriculant with an ATOMS score of 80 would be at the 61st percentile rank. If the gender is unknown, then the percentile rank on the norm table for men and women combined can be used to estimate.

**Table 3:** Comparisons of Scores of the Attitudes Toward Osteopathic Medicine Scale (ATOMS) by Gender, Race/Ethnicity, Academic Background and Career Interest in a National Sample of First Year Matriculants of 33 U.S. Colleges of Osteopathic Medicine

Variable		N	M	SD	F-ratio	p
Gender^(a)^						
	Men	2653	71.5	10	F_(__1,5615)_ = 362.31	p<.0001
	Women	2964	76.1	8.3		
Race/Ethnicity^(b)^						
	White/Caucasian	3386	73.94	9.4	F_(__3,5099)_ = 15.80	p<.0001
	Asian	1315	72.88	9.5		
	Hispanic/Latinx/ Spanish origin	235	75.85	8.4		
	Black/African American	167	77.27	9.2		
Academic Background ^(c)^						
	Biological Sciences	2833	74.12	9.3	F_(__3,3939)_ = 13.04	p<.0001
	Chemical/Physical Sciences	755	71.8	9.7		
	Social/Behavioral Sciences	264	74.52	9.7		
	Arts and Humanities	91	74.35	9.9		
Career Interest^(d)^						
	People-oriented	2347	75.17	9	F_(__2,5110)_ = 40.21	p<.0001
	Technology- oriented	1542	72.55	9.9		
	Other	1224	73.25	9.6		

^(a)^Women > Men, effect size= 0.51.

^(b)^ Black/African American > Hispanic/Latinx/Spanish origin > White/Caucasian and Asian, effect size = 0.47

^(c)^Chemical/Physical Sciences < all others, effect size = 0.28

^(d)^People-oriented > Other > Technology-oriented, effect size = 0.28

## Discussion

Using a nationwide sample, we developed and validated an instrument to measure students’ orientations toward osteopathic medicine. Moreover, the study allowed us to prepare national norms that will enable medical colleges to determine the percentile rank of first-year matriculants in U.S. osteopathic medical colleges. More importantly, in all statistical analyses, we controlled for the effect of social desirability bias by excluding those who attempted to give a “good impression” response and scored above the cutoff of the Infrequency scale of the Zuckerman-Kuhlman Personality Questionnaire.^27^

This study is unique, because to our knowledge, with the exception of studies in which we retrieved data from the Project in Osteopathic Medical Education and Empathy (POMEE), no other published study in medical education has been undertaken in which a large nationwide sample of medical students participated, and in which the social desirability response bias, which is a shortcoming of self-reported personality tests, was controlled.

Our findings on the magnitude and direction of item-total score correlations indicate that items of the ATOMS contribute significantly and positively to the total score. Cronbach’s alpha coefficient of 0.83 for the ATOMS scores is in the acceptable range for psychological and educational tests. The large magnitude of effect sizes of item discrimination indices confirms the ability of ATOMS items to discriminate between students with the most favorable and the least favorable attitudes toward osteopathic medicine. The large magnitude of effect sizes indicate that the difference in mean item scores between high and low scorers in favor of high ATOMS scorers were not only statistically significant but also practically (clinically) important.^32^

The three underlying factors of the ATOMS that emerged from factor analysis not only corroborate the face and content validity of the instrument but also made it possible to recognize and quantify core components of orientation toward osteopathic medicine. The criterion-related validity of the ATOMS scores was supported by statistically significant and positive correlations with scores of conceptually relevant measures. In particular, higher correlations with scores from the orientation toward interprofessional collaboration and clinical empathy (conceptually more relevant to competencies of osteopathic medicine) support the “convergent” validity of ATOMS scores. Conversely, lower correlations with measures of attitudes toward lifelong learning and affective empathy (conceptually less relevant to core tenets of osteopathic medicine) support the “discriminant” validity of ATOMS scores.

Patterns of findings in the expected direction obtained by using the method of contrasted groups provided additional evidence in support of the validity of the ATOMS scores. Because of the significant correlation found between ATOMS and clinical empathy (JSE) scores, we expected to find group differences similar to those in our previous research on empathy. For example, the ATOMS mean score was significantly higher for women than for men, a pattern of difference observed for clinical empathy in allopathic^33^^,^^34^  and osteopathic medical students.^31^ Also, group differences in ATOMS scores by race/ethnicity was consistent with previous findings regarding JSE scores in osteopathic medical students.^31^ Similarly, differences in ATOMS scores by academic background were consistent with previous findings regarding JSE scores among osteopathic medical students.^31^ Group differences in ATOMS scores by career interest were consistent with previous findings regarding JSE scores among allopathic^33^ and osteopathic medical students.^31^  

The inverse relationship between ATOMS scores and burnout scores also supports the validity of ATOMS scores, consistent with other studies.^35^ Additional research is needed to explain the difference in the direction of correlation between ATOMS scores and clinical empathy scores as opposed to emotional empathy scores. Perhaps the cognitive nature of clinical empathy (measured by the JSE) as opposed to affective nature of emotional empathy (measured by the subscales of the IRI) could explain their corresponding positive and negative correlations with the ATOMS scores.

A limitation of the findings is that national norms developed for new matriculants cannot be used for students in different years of medical school unless further empirical evidence verifies that ATOMS scores do not significantly change as students progress through medical school, which seems unlikely, based on previous findings.^6^^,^^30^

**Table 4:** National Norm Table for the Attitudes Toward Osteopathic Medicine Scale (ATOMS) in a National Sample of First Year Matriculants from 33 U.S. Colleges of Osteopathic Medicine

ATOM	Men (n=2653)			Women (n=2964)			Combined (n=5617)		
Raw Scores	f	cf	%Tile Rank	f	cf	%Tile Rank	f	cf	%Tile Rank
≤ 46	17	17	<1	1	1	<1	18	18	<1
47-48	14	31	1	1	2	<1	15	33	<1
53-54	46	154	5	10	42	1	56	196	3
55-56	65	219	7	18	60	2	83	279	4
57-58	75	294	10	25	85	2	100	379	6
59-60	99	393	13	42	127	4	141	520	8
61-62	109	502	17	74	201	6	183	703	11
63-64	119	621	21	84	285	8	203	906	14
65-66	157	778	26	97	382	11	254	1160	18
67-68	183	961	33	135	517	15	318	1478	23
69-70	186	1147	40	172	689	20	358	1836	29
71-72	230	1377	48	248	937	27	478	2314	37
73-74	220	1597	56	256	1193	36	476	2790	45
75-76	212	1809	64	259	1452	45	471	3261	54
77-78	181	1990	72	238	1690	53	419	3680	62
79-80	146	2136	78	249	1939	61	395	4075	69
81-82	148	2284	83	251	2190	70	399	4474	76
83-84	89	2373	88	279	2469	79	368	4842	83
85-86	111	2484	92	217	2686	87	328	5170	89
87-88	94	2578	95	151	2837	93	245	5415	94
≥ 89	75	2653	99	127	2964	98	202	5617	98

f: Frequency, cf: Cumulative Frequency.

Respondents who did not specify their sex as “male” or “female” (< 1%) were excluded in this table.

The limitation regarding self-reported measures of personal attributes, influenced by social desirability response bias, could be mitigated by using the Infrequency Scale of the ZKPQ to control for “good impression” response bias.

## Conclusions

The ATOMS instrument, developed and validated in this study, benefits from strong psychometric support and brevity. It has important implications for assessing osteopathic educational outcomes (e.g., examining improvement in ATOMS scores as students progress through medical school, or exposed to a targeted educational program/workshop to enhance their understating of the philosophy and tenets of osteopathic medicine), and patient outcomes (e.g., associations between physicians’ ATOMS scores and positive patient or clinical outcomes). Furthermore, the national norms developed in this study have implications for admissions decisions (e.g., to recruit students who are more inclined to embrace the osteopathic philosophy, or simply to break the tie between applicants with similar academic credentials). Because of sound psychometric support (e.g., face, construct, criterion-related validity, and internal consistency reliability), brevity, unique advantage in using nationwide sample, and control for social desirability response bias, and because of visibility of POMEE in osteopathic medical education community, we anticipate broad use of the ATOMS in future osteopathic medical education research.

### Acknowledgements

We would like to acknowledge those who have contributed significantly to Phase II POMEE including the research coordinators who served as liaisons between their college and research teams at AACOM and Jefferson. They are: Michael Becker, DO, MS; Joseph Brewer, PhD, MD; Carla Case; Mark Clark, PhD; Karen Clayton, PhD; Robyn Dreibelbis, DO; Glenn Davis, MS; Kyle Henderson, PhD; Selma Holden, MD; Ana Maria Homs, PsyD, MBA; Justina Hyfantis, PhD; LeAnn Jons-Cox, DO; Schoen Kruse, PhD; Lisa Carroll, MD; Gretchen Lovett, PhD; Susan Mackintosh, DO, MPH; Patience Mason, MEd; Elizabeth McClain, PhD; Eric McLaughlin, MS; Terrence Miller, PhD; Luke Mortensen, PhD; Bruce Newton, PhD; Edward Magalhaes, PhD, LPC; Nagesh Rao, PhD; Lorree Ratto, PhD; Sean Reeder, DO; David Roos, EdD; Raquel Romanick, JD; Katherine Ruger, EdD; Vivian Stevens, PhD; Patricia Sexton, MS, DHEd; Vivian Stevens, PhD; Mary Ann Taylor, PhD; Clinton Whitson, MS; Rynn Ziller, EdD. Also, thanks to Aaron Douglas, PhD, and Bruce Newton, PhD, for their valuable comments; Shira Carroll, administrative assistant at Jefferson, for managing administrative aspects of the project in addition to editorial and stylistic modifications of the manuscript; and Pamela Walter for her editorial polishing help. We thank Dr. Craig D. Schneider for granting us permission to adapt and use 7 items from the Integrative Medicine Attitude Questionnaire, Dr. Mark Davis for granting us permission to use two scales of the IRI, and Dr. Samuel Melamed for his permission to use his burnout scale. We are also thankful to the deans of participating colleges of osteopathic medicine who notified and encouraged the study cohort in their schools to participate and continue their cooperation in the project, and finally we thank thousands of osteopathic medical students who participated in this study and completed the study survey.

#### Funding support

This study was funded by the American Association of Colleges of Osteopathic Medicine (AACOM) and cosponsored by the American Osteopathic Association (AOA). The funding was terminated at the end of the 2019-2020 academic year, due to changes of leadership in the AOA and shift of research priority.

## Conflict of Interest

The authors declare that they have no conflict of interest.

## Supplementary material

Supplementary file 1 Appendix A. Attitudes Toward Osteopathic Medicine Scale (ATOMS)
