# Supplementary material for: Attitudes toward osteopathic medicine scale: development and psychometrics
Source: Int J Med Educ. 2021 Nov 19;12:222–32. doi: 10.5116/ijme.615c.2cfa (PMC12928722; doi:10.5116/ijme.615c.2cfa)
Supplement: Supplementary file 1 — Appendix A. Attitudes Toward Osteopathic Medicine Scale (ATOMS) [file ijme-12-222-S1.pdf]

Appendix A

Attitudes Toward Osteopathic Medicine Scale (ATOMS)

Instructions: Please indicate the extent of your agreement or disagreement with each of the following statements by checking the appropriate circle, using the following 7-point scale (a higher number indicates more agreement).

1-------2-------3-------4-------5-------6-------7

Strongly Disagree *Strongly Agree*

Statement	①②③④⑤⑥⑦
1. The osteopathic philosophy of holistic care greatly influenced my decision to attend an osteopathic school.	○○○○○○○
2. Patients whose physicians are knowledgeable of multiple medical systems and complementary and alternative practices, in addition to conventional medicine, do better than patients whose physicians are only familiar with conventional medicine.	○○○○○○○
3. Therapeutic touch has been discredited as a healing modality.	○○○○○○○
4. Physicians with a balanced lifestyle (i.e., attending to their own health, social, family and spiritual needs, as well as interests beyond medicine) generate improved patient outcome.	○○○○○○○
5. Osteopathic Manipulation often makes patients "feel" better temporarily but does not lead to objective improvement in long-term outcomes for patients.	○○○○○○○
6. A strong relationship between patient and physician is an extremely valuable therapeutic intervention that leads to improved outcomes.	○○○○○○○
7. Instilling hope in patients is a physician's duty.	○○○○○○○
8. Osteopathic manipulative therapy is a valuable method for resolving a wide variety of musculoskeletal problems (beyond back pain).	○○○○○○○
9. Information about the relative effectiveness of treatments that is obtained by research methods other than randomized controlled trials has little value to physicians.	○○○○○○○
10. Physicians who strive to understand themselves provide better care than those who do not.	○○○○○○○
11. Psychosocial factors are as important as biomedical factors in health and illness.	○○○○○○○
12. Medical problems need specific medical and surgical interventions; thus, holistic approaches to medical problems cannot be as beneficial as targeted biomedical treatment.	○○○○○○○
13. Touch and tactile approaches may not serve a significant purpose in patient care.	○○○○○○○

©American Association of Colleges of Osteopathic Medicine, 2021.
Interested researchers are permitted to use the ATOMS in their not-for-profit research, as long as the instructions to complete the test, text, and order of appearance of items, and the 7-point response scale remain intact. In addition, recommended scoring algorithm must be used, and appropriate credit must be given to the original source. Address all inquiries about the ATOMS and its scoring algorithm to Mohammadreza Hojat, Ph.D. (mohammadreza.hojat@jefferson.edu
